# M. Cazenave on Diseases of the Skin

**Published:** 1832-10-01

**Authors:** 


ABRÉGÉ PRATIQUE DES MALADIES DE LA PEAU, D'APRES LES AUTEURS LES PLUS ESTIMÉS, ET SURTOUT D'APRES DES DOCUMENS PUISÉS DANS LES LECONS CLINIQUES DE M. le Docteur *Biett.* Par *A. Cazenave* et *B. E. Schedel,* Docteurs en Medecin. A Paris, 1828.

[Continued from Page 407, Vol. XVI. (April, 1832.)]

### MACULÆ.

THIS order comprehends those changes in the colour of the skin depending on an alteration in its pigment. They are seated in the rete mucosum, and are divided into those characterized by an increase of colour, and by a deficiency. The former are either general or partial. The bronze tint of the skin, is the only example of general discolouration. It has particularly been observed to follow the internal use of nitrate of silver, but cases have occurred whose origin could not be traced to the employment of this medicine; in these the colour is less deep, and the skin is of a dirty rather than a bronze hue. The discolouration arising from the long-continued use of nitrate of silver is at first blueish, this gradually turns to a light bronze, which is most visible on those parts exposed to the sun's rays, and where the skin is thin, as the face and hands. The whole surface of the body is discoloured at the same time; gradually the tint becomes deeper, until in some cases it is almost black. The conjunctivæ are generally of a livid coppery colour, as well as the mucous membrane of the mouth, where it is exposed to the light, at its junction with the skin. It is remarkable that the bronze colour of the face becomes darker under the influences of those causes which generally produce paleness, and lighter when the individual is exposed to any cause which under ordinary circumstances would render the face red. This discolouration is probably permanent; M. Biett has seen, in Geneva, two individuals in whom it has remained more than twenty years with the same degree of intensity, and during the last fourteen years, which time he has used nitrate of silver in his own practice, he has observed the same thing in several cases. In some the colour has slightly diminished, but hitherto no instance has been known in which it has completely disappeared. It is not attended with any general symptoms, or disturbance in the economy. The nails are usually blueish, but the hair is not changed at all in colour. Generally the cicatrices which existed previously to the discolouration participate in the bronze hue, but M. Biett has observed that those which have formed subsequently remain white, particularly if the wound was deep. Foucroy was the first who attracted attention to this important change in the colour of the skin. Subsequently cases have been recorded by Powell, Marcet, and Roget in England, by Albers, Reimar, and Schleiden in Germany, and by Butini, Delarive and Odier in Switzerland; M. Biett has seen in Paris 22 cases, 7 of which were females, 15 men. The influence of nitrate of silver on the secretion of pigment has not yet been satisfactorily explained. No treatment appears to produce any effect on this discolouration; M. Biett has recommended to two patients whom he has attended nearly fourteen years, salt water baths, as well as baths containing alkalis or the various salts of iron, but without obtaining the least change. He has applied blisters to the hands without effect. Probably reiterated blistering might diminish considerably the dark bronze hue, particularly if the blisters were constantly renewed on the denuded surface, but the difficulty of applying such a remedy to the various elevations and depressions of the face would be insurmountable, and the piebald appearance of the disfigurement but partially remedied, would be more disagreeable than the uniformly unnatural tint.

*Lentigo (Ephelide lentiforme. Alibert), Freckles.* This is characterized by small rounded yellowish spots, scattered irregularly, sometimes uniting, particularly about the nose and cheeks, and forming large patches; in many cases they are congenital, in others they are developed about the ninth or tenth year; they remain during life, but are more evident during certain periods. They are more marked in youth, affecting the face, hands, neck and parts of the chest, but in some cases the whole surface has been spotted with them. These maculæ affect chiefly those of a lymphatic habit, whose skin is thin, white, and delicate; rarely the strong and sanguineous. They are sometimes caused by exposure to the sun's rays; in such cases they disappear on changing the climate, or by time. Lentigo is never accompanied with any disturbance in the health, nor does it demand any treatment.

*Ephelides (Pityriasis Versicolor. Willan.)* These are irregular patches, much larger than the spots of lentigo, of a saffron yellow colour, frequently accompanied by itching, and giving rise sometimes to a slight exfoliation. They generally are seen on the anterior part of the neck and breast, on the breasts of women, on the abdomen, groins and internal surface of the thighs. They rarely affect the face, except in women who are pregnant. Most frequently they are slowly developed and remain many weeks, or even months, if they are not subjected to medical treatment. Occasionally they appear a short time before the menstrual discharge and disappear as soon as that evacuation is established. They are not accompanied by any constitutional symptoms, but they cause a troublesome local itching, which is augmented by the slightest moral impressions or irregularities in diet; it is increased in females about the time of menstruation, and aggravated by their scratching the part, a practice which they can rarely resist.

*Causes.* They affect more particularly women whose skin is pale and thin, occasionally however individuals are liable to them whose skin is brown, and hair black, they are then of a deeper colour. They are sometimes caused by exposure to the sun, by irregularities of diet, salt or smoked food, or by the suppression of habitual evacuations, as the menses and hemorrhoids; in the majority of cases those who are the subjects of them are in good health.

*Diagnosis.* From pityriasis by the exfoliation being slight and powdery, not a desquamation of small scales of altered epidermis. Pityriasis is never accompanied by that constant itching which attends ephelis. The livid or copper-coloured hue, the absence of exfoliation, and of all itching, the history of the case, and often the concomitant symptoms, distinguish syphilitic blotches from this form of cutaneous discolouration.

*Prognosis.* Those which appear on the face at the commencement of pregnancy, remain perhaps only a few months or until delivery, they require no treatment; those which precede or accompany the menstrual periods are very ephemeral. Those occurring under other circumstances, produce no other inconvenience than a troublesome itching, and yield readily to an appropriate treatment.

*Treatment.* Astringent lotions, alkaline ointments, &c. are useless. The treatment which is most successful is, two or three sulphur baths in the week, and the internal use of the sulphurous waters, as those of Eughien or Cauterets, diluting the former at first with milk or barley-water. If the itching is insupportable, the patient may make use of a lotion consisting of an ounce of sulphuret of potash dissolved in two pints of water, on the days on which he omits the bath.

*Nævi Materni.* Our authors have not attempted to explain the causes of these curious productions, nor have they suggested any mode of removing them. Bateman, in his Synopsis, gives a much more full and complete account of their various forms; and in the article "Imagination," in Rees's Cyclopædia, he has, in an amusing and instructive article, discussed the question as to the share which the imagination of the mother has in the formation of these discolourations. There are two genera in this order characterized by deficiency of colouring matter; one general, or albinism—the other partial, vitiligo. As there is nothing new or of practical importance in the description of the former, we shall pass on to the latter.

*Vitiligo.* This may be a congenital or an accidental formation. The congenital has only been observed among the negroes who have occasionally been marked with various white spots, and consequently called "pied negroes." The accidental variety is most frequently seen in the scrotum, particularly of old people, in the form of irregular milk-white patches or longitudinal streaks; they are neither attended with heat, nor itching. They may increase to a very considerable extent. This disease has never been benefitted in the Hôpital St. Louis by M. Biett, who has tried various remedies. Bateman describes vitiligo under tuberculæ; he says that it commences by white shining tubercles, which in the course of a week attain their full size, that of a large wart, and then begin to subside, becoming flattened to the level of the cuticle in about ten days; in other cases they are less distinctly tubercular and more permanent. Of course Dr. Bateman did not describe this from imagination; the discrepancy between the two descriptions may be accounted for, by supposing that our authors have not seen the early stages of the eruption: in the few cases which we have seen, there certainly was no appearance of tubercles, but merely slightly elevated smooth white patches; but the complaints were of some months' standing.

### Diseases of the Skin which cannot be referred to either of the preceding Orders.

*Lupus (Herpes exedens—Dartre rougeante, Alibert.)* Lupus commences sometimes under the form of reddish violet spots, but generally by livid, indolent tubercles, of various sizes. It is characterized by its tendency to destroy the surrounding skin and subjacent tissues, by means of unhealthy ichorous ulcers, covered with brownish adherent crusts. It most generally attacks the face, and the nose is the part most exposed to its destructive ravages, but it may affect the body and limbs; on the trunk it is observed, especially on the breasts and shoulders; when it affects the limbs, it is situated near the articulations, on the external surface of the fore-arm, or the back of the hand and foot; it sometimes is seen on the neck. It may be confined to one of these situations, or it may at the same time, or progressively, attack several of these parts of the body. Lupus, in the greater number of cases, commences by small, dull red, hard, indolent tubercles, appearing to affect only the most superficial layers of the skin; sometimes their summit is covered with small white and dry scales; frequently many tubercles unite, forming a surface of greater or less extent, not painful, soft to the touch, and finally ulcerating. But lupus does not invariably commence with tubercles; sometimes it begins with inflammation of the mucous membrane lining the nasal fossa, accompanied with redness and swelling of the nose; a thin scab forms there, this is removed, and is immediately replaced by a thicker incrustation, for the destructive process has commenced. In other cases, some part of the face, especially the end of the nose, becomes of a violet red colour, and slightly tumefied; this remains many months; the colour gradually increases, the surface becomes more vascular and ulcerates; finally, the skin may gradually become thinner and livid, looking like a cicatrix, but neither preceded by tubercles nor ulceration. For these reasons, M. Biett has not, in this instance, followed the classification of Willan, who placed lupus among "tuberculæ." He has divided the affection into three varieties; the division is wholly practical, and tends to faciltate both the study and the description of this formidable malady. 1. Lupus destroying the surface. 2. With deeply-penetrating ulceration. 3. With hypertrophy.

1. *Lupus destroying the Surface.* In some very rare cases, it seems to affect only the most superficial layers of the skin; this variety is observed on the face, especially on the cheeks; it neither commences with tubercles nor with ulcerations; the skin grows red, the epidermis exfoliates, the skin becomes gradually thinner, it is shining and smooth like the cicatrix of a superficial burn; the patient experiences no pain except on pressure, under which the redness disappears. After violent exercise, or excess in drinking, the surface becomes sensible. When the disease ceases to make any progress, the redness disappears as well as the exfoliations of the epidermis, but the skin remains thin, smooth, and shining. In other cases, small, soft, dull red tubercles form; after remaining some time stationary, they suddenly increase in size and number; the skin between them becomes swollen and œdematous, their bases unite, their summits ulcerate, and speedily the whole surface is covered with an irregular unhealthy ulceration, having a blackish, very adherent scab; this increases in extent in one direction, frequently healing and forming white, irregular cicatrices in another, especially if suitable treatment has been employed. In this way it gradually may extend, and even cover the whole face; in bad cases new tubercles form on the old cicatrices, which are soon involved in the general ulceration; as this process extends by means of the formation of new tubercles the ulceration is always bounded by a hard, rough, and tumefied border. The nose, which is rarely the primitive seat of this variety, becomes involved in the ulceration, and often, on the scabs falling off, part of the alæ nasi, or of its extremity, is found to have accompanied them. When the scabs have been removed and a suitable treatment has been adopted, they are not renewed, the surface becomes covered with small thin scales, and a solid white cicatrix forms in many points. This variety may occupy large surfaces of the breast, limbs, anterior part of the thighs, &c.

2. *Lupus with deeply-penetrating Ulceration.* This variety occupies particularly the alæ and the extremity of the nose; in the majority of cases its appearance is preceded by redness, swelling and coryza. One ala becomes swollen and painful, of a violet red colour, it slightly ulcerates, a small scab is formed, which on falling off is replaced by a thicker, each time this is attended by a true loss of substance, slight at first, but eventually very striking. The ulceration extends to the opposite side of the nose, and gradually increases, but is attended with but little pain; the cartilages are destroyed, and on removing the scabs an unhealthy ulceration is exposed, which discharges an abundant sero-purulent fluid. There is often a fetid discharge from the nose, and so considerable a tumefaction that the destruction is not recognized until the swelling subsides. In other cases there is neither coryza, nor tumefaction; a small, red, smooth and soft tubercle forms, which eventually ulcerates. The extent of the destruction is variable, sometimes the whole nose and its septum are destroyed, or only its extremity; often the surface of the nose only has been destroyed, so that it is generally diminished in volume and more pointed, the nostrils having a constant tendency to close; it is constantly red, except along the angle of the junction of its two lateral portions, where the projecting cartilage, of a yellow colour, is plainly seen through the transparent cicatrix. In other cases, a portion of the nose appears to have been cut off with a knife. The extent of the destruction is not in relation with the duration of the disease; sometimes after many years but a small portion of the nose is destroyed, whilst the whole organ in other cases has been eaten away in 10 or 15 days. In almost all cases of lupus of the nose there exists at the same time an affection of the mucous membrane of the nasal fossæ, and even the whole septum narium has been destroyed before the nose has suffered externally. At other times the ulceration is continued from the skin to the pituitary mucous membrane; it creeps along the floor of the nasal fossæ and attacks the mucous lining of the palate and the gums themselves. The disease may extend itself from the nose to other parts of the face.

3. *Lupus with Hypertrophy.* This commences almost exclusively in the face, to which it is confined, by soft, indolent, and but slightly elevated tubercles, generally numerous; they occupy a considerable surface, such as a great part of the cheek, and sometimes the whole face; they do not ulcerate, or at least the ulcerations that are sometimes seen are rare and almost accidental; gradually the base of these tubercles enlarges, the skin and subjacent cellular tissue become the seat of an indolent tumefaction, after some time the face is spotted with reddish points, which are the tubercles brought by the tumefaction of the subjacent tissues to the level of the skin; among them are numerous white spots or true cicatrices, which have replaced the old tubercles, although these have not been previously destroyed by ulceration; in fact, these tubercles constantly exfoliate and are gradually destroyed. In such circumstances the face may acquire a prodigious size, the cheeks are soft and flabby, their tissue resembling that of elephantiasis; the forehead and eyelids are so tumefied, that the eyes are almost entirely covered with these hypertrophied masses; the lips and ears participate in this general enlargement. This disease may persist for an indefinite period; when the parts begin to recover their natural state, which they never do spontaneously, their circulation is increased, slow resolution of the tubercles takes place, and the hypertrophy gradually diminishes, but hardly ever wholly subsides. There is another variety of lupus with hypertrophy, in which the ulcerations which succeed either to the violet spots or to tubercles become covered with soft red, prominent, and small fungous tumours. This is a very severe form.

These three varieties of lupus may exist simultaneously in the same individual; thus the first may cover one part of the face whilst the nose is being destroyed by the second, or whilst the other cheek is the seat of the third. In some cases, the variety attended by superficial ulcerations may be accompanied by hypertrophy; in some instances these ulcerations destroy the lower eyelid, so that the skin of the cheek is directly continuous with the conjunctiva covering the eye; this membrane, from constant exposure, becomes inflamed, and eventually opacity of the cornea produces complete blindness; in other cases partial destruction of the inferior lid is the cause of various forms of ectropium. In other circumstances, on the removal of the thick and long-adherent incrustations, the nostrils will be found to have been completely obliterated by the hypertrophy or by neglected cicatrices. In a similar manner, the size of the mouth is occasionally considerably diminished. Notwithstanding the destructive effects of this disease the general health is not affected, the patients enjoy a good state of health; the menstrual discharge, alone, in some women seems to be deranged. Erysipelas of the face very frequently complicates this affection, and generally, instead of aggravating it, is beneficial. It has in many cases, especially of lupus with hypertrophy, increased the vitality of the skin so as to produce a speedy resolution. Finally, in some of the most destructive cases where the lupus successively destroys the skin, the cartilages, and the bones, symptoms of chronic gastro-enteritis come on, and a slow fever with diarrhœa destroys the patient. This unfortunate termination is extremely rare. The bones are very seldom destroyed by lupus; many instances have occurred in the Hôpital St. Louis of patients who have been affected with this disease for many years, without any energetic treatment having been employed, and very rarely has any destruction of the osseous system occurred, excepting the loss of the ossa nasi, which bones have frequently disappeared, leaving a triangular opening divided by what remains of the septum-narium.

*Causes.* Lupus affects especially children and adults, it has been very rarely observed after the fortieth year; it attacks both sexes, and nearly in the same proportions, and those living in the country seem more disposed to it than the inhabitants of towns; this may perhaps depend on bad food and unhealthy localities. Very frequently it commences in scrophulous children and remains until puberty; it can only be said to co-exist with scrophula, as many of the individuals who are attacked with it are robust, healthy adults. The form of lupus with hypertrophy is especially connected with a highly scrophulous constitution.

*Diagnosis.* The circumscribed indurations which succeed the pustules of acné rosacea might be mistaken for the tubercles of lupus in an early stage; but the red colour, the erythematous areolæ, the pre-existence of pustules, and their frequent presence around the part are sufficient marks to distinguish acné, from the livid, indolent tubercles of lupus, which were preceded only by a light violet stain. Lupus with hypertrophy may be confounded with the tubercular elephantiasis (E. Græcorum), but in elephantiasis the skin is yellow, there are small tuberculated and unequal tumours attended with partial hypertrophy of the integuments of the face differing from the uniform and equal tumefaction of lupus; if the tubercles have ulcerated, the ulcerations of elephantiasis G. will be found to be more superficial and to have no tendency to attack the healthy parts. Elephantiasis of the Greeks in general exists in many parts of the body at the same time, and in this stage is attended with such general and local symptoms as at once distinguish it from lupus. On a superficial examination the incrustations of lupus may be taken for the scabs of impetigo, but the latter are yellow, rough, prominent, slightly adherent; the former brownish, thick and very adherent, besides attention to the elementary lesions, and to the cicatrices and ulcerations of lupus will alone be sufficient to establish the diagnosis. There remain two other diseases which are often much more difficult to distinguish from lupus, and whose diagnosis is highly important, these are noli me tangere, and some varieties of syphilitic eruptions. M. Biett applies "noli me tangere" to true cancerous affections of the face which have been confounded with lupus. Cancerous tubercles are hard, generally indolent, and painful, appearing in advanced life either on the lips, cheeks, or nose, where they remain a considerable time without ulcerating; lupus hardly ever is observed in individuals of advanced life, it usually commences with numerous tubercles, noli me tangere by a solitary one; the tubercles of lupus are situated in the superficial layers of the skin and are constantly indolent, whilst cancerous tubercles are surrounded by a hard and circumscribed base, and are usually the seat of very acute lancinating pains. Noli me tangere is accompanied by inflammatory tumefaction of the soft parts, it is usually exasperated by cauterization, and when once ulcerated it not only destroys the skin and cartilage of the nose but the bones to a considerable depth; this is never observed in lupus. Cancerous ulcerations are moist, painful, with everted edges; they have a fungous appearance, and are not covered with dry thick incrustations. Syphilitic tubercles may be mistaken for those of lupus; but the former are rounder and more voluminous than those of lupus; their summits never exfoliate, nor are they so prone to ulceration; syphilitic tubercles of the face are never found in children. The ulcers which succeed syphilitic tubercles are deep, their edges are tumefied, perpendicular and of a coppery red, whilst those following the tubercles of lupus are of a dull red, and are superficial. Lupus with deeply penetrating ulceration is more likely to be confounded with syphilitic ulcers, particularly when the nose is destroyed; the mode in which this destruction takes place distinguishes them. Thus, in lupus, the skin is at first destroyed, the cartilages and the ossa nasi subsequently; in syphilis, at least under these circumstances, the disease has commenced in the bones, these have become carious, and the soft parts then ulcerate. The constitutional symptoms of secondary syphilis afford another means of diagnosis.

*Prognosis.* This is always unfavourable, not from lupus endangering the life of the patient, but from its intractable nature, its destructive progress, and the indelible and deforming cicatrices which remain even when it has been cured. As long as the cicatrices remain soft and blueish, and on pressure give the feeling of fluctuation, and as long as they are circumscribed by tubercles, the return of the disease is to be feared.

*Treatment.* General treatment may be of service if the patient is scrofulous: in such cases some advantage may be derived from the internal administration of muriate of lime, in the proportion of a drachm to a pint of water; a spoonful of this may be taken every morning for five or six days, it may then be gradually increased until the patient takes 12 or even more spoonfuls daily; the patient should be put on generous diet, and live where he can breathe a pure air. In other circumstances, to hasten the resolution of the tubercles, active remedies have been employed, which, together with judicious local treatment, have sometimes contributed to the cure, such are, the animal oil of dippel given at first in doses of five or six drops, and gradually increased to twenty-five; the asiatic pills; Pearson's solution of arsenic, in scruple doses, gradually increased to a drachm, or Fowler's solution, (liq. arsenicalis,) commencing with four or five drops daily, and gradually augmenting the dose to twenty or twenty-five drops. Attention should be paid to diet, &c. and exposure to extreme cold scrupulously avoided, as well as excessive heat. The local treatment consists, 1st, in applications whose object is to modify the vitality of the skin, so as to produce resolution of the tubercles; 2d, in caustics, to change the state of the morbid surface, to circumscribe its ravages, and to obtain firm cicatrices. Those applications intended to produce resolution of the tubercles, are to be applied before these have ulcerated, and to those which surround cicatrices; they are especially suited to lupus with hypertrophy. The most useful are ointments, consisting of one scruple or half a drachm of the proto-ioduret of mercury, or of twelve or fifteen grains or a scruple of the deuto-ioduret to an ounce of lard;—slight frictions are to be made with these ointments on all the points which are covered with tubercles. Twelve or fifteen grains of the ioduret of sulphur to an ounce of lard is a very energetic and successful remedy. In some cases these remedies have appeared to hasten the ulcerative process; if this has commenced, cauterization must be employed. If the disease is extensive, the cautery should be applied at first to a small part of the surface, not at once to the whole disease. If the surface is ulcerated, moist and clean, the cautery may be directly applied; but if there are scabs, these must be previously detached by poultices: lastly, if the tubercles are not ulcerated, but are violet, dry, and accompanied by more or less of tumefaction of the skin, or if it is a case of lupus with hypertrophy, to which it is proposed to apply this remedy, the skin should be destroyed previously by blisters. The animal oil of dippel acts less as a caustic than an irritant, which sometimes modifies the action of the part to which it is applied very advantageously, but it rarely produces a complete cure. It is particularly suitable where the nose is the seat of an indolent and chronic swelling, of a violet colour, with a constant exfoliation of the epidermis. In order to apply it, a small camel's hair-pencil may be dipped in this liquid, and the diseased surface frequently smeared with it. Cauterizations with nitrate of silver, potash, and butter of antimony, succeed less frequently than the following applications. The powder of Dupuytren, consisting of one or two grains of arsenious acid and 98 of calomel, is a very useful and mild caustic; it is suitable in lupus of slight extent affecting children, women, or irritable men; the sore is to be sprinkled with this powder, by means of a little puff; any crust which might have covered the surface being first removed by a poultice, or by other means. Although the application of this caustic is not generally followed by pain or swelling of the surrounding parts, it is better not to apply it each time to a surface much larger than a shilling; it forms a greyish, very adherent incrustation, which does not fall off for a very considerable time, unless emollient applications are used. The arsenical powder du frère Côme is a more valuable and energetic remedy, and requires to be employed with prudence. It is suitable in those cases where less active applications fail in stopping the destructive progress of the disease, and particularly to that variety of lupus which is attended with deeply-penetrating ulceration. This liquid paste should be applied to a surface not so large as that of a shilling, by means of a spatula. This is constantly followed by erysipelas, sometimes very slight, at other times severe; but, although our authors have seen it applied by M. Biett in the wards of the Hôpital St. Louis, a large number of times, not a single case has been attended with any dangerous symptoms; the erysipelas has yielded in a few days to leeches behind the ears, stimulating foot-baths, abstinence from food, and emollient or laxative glysters. Finally, the acid nitrate of mercury,* which is an energetic caustic, has been em-

* One drachm of nitrate of mercury dissolved in an ounce of nitric acid.—*(Formulaire des Hôpitaux, &c.)*

ployed with much success in the wards of M. Biett. It produces an erysipelatous inflammation of a less severe character than that following the arsenical paste. It may be applied not only on the ulcerated surface, but to the tubercles themselves, and to the cicatrices which remain soft, blue, and fluctuating. A surface about the size of a crown-piece (5 franc piece) may be cauterized by means of a small pledget of lint dipped in this acid, and passed over the disease; and charpie moistened in the same liquid may be applied over the cauterized part. The part becomes white, a yellowish crust forms which is detached after eight or fifteen days. This application is generally very painful, but the suffering is momentary. The actual cautery is not advisable, as it generally aggravates the disease. When the cauterization has been effectual, it is found, on the crusts becoming detached, that the ulceration beneath them has a healthy appearance and often speedily cicatrizes; but, in the majority of cases, a single cauterization is not sufficient—it requires frequent repetition, even for years, when the disease is extensive, as it will yield only to perseverance in such treatment. Great patience is requisite in the surgeon as well as in the patient. Unless care is taken, the formation of cicatrices may be attended with considerable deformity; for example, the openings of the nostrils may become permanently obliterated, unless pieces of properly prepared sponge are daily introduced;—it should be recollected that the tendency which these openings have to become closed, continues even after the ulcerations are healed. The local and general treatment of lupus will in general sometimes be advantageously seconded by the use of simple or vapour baths; the vapour douche baths are particularly useful in lupus with hypertrophy. We need not apologize for dwelling so long on this formidable affection, when the text-book of English practitioners for diseases of the skin contains its description in less than a dozen lines with this excuse—" of this disease I shall not treat at any length, for I can mention no medicine which has been of any essential service in the cure of it."—*(Bateman's Synopsis, p.* 408, 7*th edition.)* The description given by Mr. Plumbe is evidently derived from a very limited number of cases, and is deficient in diagnosis.

*Syphilitic Eruptions. (Syphilides—Alibert.)* Mr. Carmichael was the first in this country who applied the exact and descriptive nomenclature of Willan to the various forms of venereal eruptions. Previously to the publication of his Essay on the Venereal Disease, they were described under the name of copper-coloured blotches, scabby spots, and other terms quite as indefinite. John Hunter described but one form, that of scaly blotches terminating in ulceration; Dr. Willan described, with his usual accuracy, syphilitic lepra; and Dr. Bateman syphilitic ecthyma. Alibert recognized more numerous varieties and formed them into a class which he denominated "syphilides," including as distinct varieties not only the tubercular, pustular, squamous, and papular forms, but the various kinds of ulcerations, the sequelæ of these eruptions: this would have been a difficult task for a man with a less vivid imagination than M. Alibert; but it must be allowed that, although he was tramelled by a most confusing classification, and has misplaced sadly his sentimental reflections, yet that his descriptions are clothed in language which for accuracy, ease, and elegance, has rarely in medical writings been surpassed. M. Biett, who has made these eruptions his particular study for many years, has been in the habit of giving clinical lectures on the whole group, and under the class "syphilides" has arranged the different varietes according to the system of Willan, by their primary external characters. This may be very convenient in a limited course of lectures, and it may afford many advantages to the student, by presenting him at one view with these hitherto imperfectly described, but most important forms of cutaneous diseases. But such a division in a systematic work like that before us, which professes an arrangement founded on external characters, and which severely criticises the attempt of Mr. Plumbe to form a classification on the supposed causes of these diseases, is open to disapprobation. We will quote our authors' own words, that our readers may judge whether their unnecessarily harsh criticism does not, in this instance, equally apply to themselves. "M. Plumbe qui a voulé adopter cette classification bizarre dans un ouvrage publié recemmement, aurait plutôt ajouté de nouvelles difficultés à celles qui existent déjà dans cette branche de la pathologie, si un ouvrage jait dans cet esprit, pouvait exercer quelque influence sur la science." We certainly do not approve of Mr. Plumbe's attempt at a new classification, but we strongly object to Messieurs Cazenave and Schedel's classing syphilitic eruptions under the anomalous cutaneous diseases which cannot be referred to any of the orders of Willan, when each variety is founded by them on the essential characters of those orders themselves.

M. Biett recognizes six forms of venereal eruptions, the exanthematous, vesicular, pustular, tubercular, papular, and squamous. All these are in general of a copper colour, less marked in the acute stage, but still never of an inflammatory red. They are almost always of a circular form; when the eruption forms considerable patches from the union of many tubercles, or pustules, &c. the circles are not complete, but small segments of circles are seen in the borders of the eruption; the scales are always thin, dry, and greyish; the crusts thick, greenish, sometimes black, always hard and grooved. These eruptions may affect any part of the skin, but they are most commonly situated on the forehead, ala nasi, back and shoulders.

1. *The Exanthematous Syphilitic Eruption.* There are two varieties; the first is primary and acute, the second consecutive and chronic. The first appears under the form of small, greyish, irregular spots of a coppery red, slightly confluent, and disappearing slowly under the pressure of the finger; it affects chiefly the trunk and limbs—it always accompanies the primitive symptoms, and especially gonorrhœa. These little spots often appear spontaneously in the course of the night; they are accompanied by slight itching, and they gradually decline in a few days, leaving a light greyish stain, which often lasts many months. The other variety is secondary; it consists of spots of a very deep copper colour, and generally exactly circular, and never confluent, nor do they disappear wholly under the pressure of the finger. They may affect the skin of the trunk and limbs, but they are very frequently found on the face and forehead; they are generally about the size of half-a-crown; sometimes, though rarely, they are covered with a slight exfoliation, and attended with itching. They are often accompanied with other syphilitic eruptions or symptoms. This eruption terminates by resolution or by slight desquamation; it never ulcerates.

2. *Vesicular Syphilitic Eruption.* This is the rarest form. M. Biett has seen it but three or four times. The vesicles are small, their base is surrounded by a red coppery areola, their progress is extremely slow, they produce no local symptom, no itching, and hardly any heat; they gradually dry up, the liquid becoming absorbed; in some it became opaque and concrete, giving rise to a small scale, which ultimately was detached; they all leave a copper-coloured stain, which has a decidedly syphilitic character. These are the appearances of the eruption in a case which is detailed, and which was the only one which had at that time fallen under the observation of our authors. Its syphilitic nature was recognized by M. Biett, who had seen two similar cases; and a decidedly syphilitic ulcer in the throat in the same patient more fully confirmed the accuracy of the diagnosis. Since the publication of this work, another case has occurred in the Hôpital St. Louis, which M. Cazenave has reported in the Journal Hebdom. Mars 14, 1829. It was complicated with syphilitic lichen, and with venereal ulcerations of the edges of the mouth. The patient was put on a restricted diet, and completely recovered without the use of mercury. Mr. Lawrence, in his surgical lectures, Medical Gazette, Vol. 5, has mentioned vesicles as occasionally accompanying other venereal eruptions, but he has neither described their peculiar appearance nor progress; we are not aware that any other English writer has alluded to them.

3. *Pustulary Syphilitic Eruption.* This may consist either of psydracious or of phlyzacious pustules. The eruption of psydracious pustules may occupy any part of the cutaneous surface, but it is most frequently found on the forehead and face; the pustules are small, extremely numerous, in groups; they are conical, and of a deep red colour; their base is hard, and surrounded by a copper-coloured areola; they appear in succession, so that they are found in different stages in the same group. Their progress is slow, but the inflammation sometimes extends deeply enough to leave a small white circular cicatrix, depressed in the centre, and of the size of a pin's head. These small cicatrices have been frequently attributed to ulcerated papulæ, from not distinguishing the elementary lesion. Psydracious pustules rarely terminate by ulceration; they dry, and form a yellow greyish crust; this leaves in some cases a cicatrix, but most frequently a stain from vascular injection. In the majority of cases the eruption consists of phlyzacious pustules, larger, generally isolated, rather depressed in the centre. Sometimes they are of the size of a lentil, numerous and but slightly prominent; their base is hard, and they contain but a small quantity of a yellowish-white purulent fluid, which contrasts strongly with the copper hue of the little tumour. They appear especially on the chest and face, and are rarely followed by ulcerations; a small crust forms, which falls off, and leaves often a cicatrix, sometimes a livid injection or a small chronic induration. It occasionally happens that numerous pustules become inflamed; they contain more purulent matter; several unite; they open, and the liquid they contained concretes into a thick greenish crust, surrounded by a large violet areola; this is always succeeded by deep ulcerations. At other times the pustules are larger, and very similar to those of ecthyma; these are generally few and distinct; they appear especially on the limbs, and chiefly on the legs, at first as a lived spot about the size of a shilling; the epidermis becomes slowly elevated by a greyish sero-purulent fluid; this is surrounded by a large copper-coloured areola; after some days it opens, and the liquid that escapes forms a very hard blackish crust, this gradually becomes very thick, and marked with circular furrows; this is not accompanied by local inflammatory symptoms; there is but little heat or pain. The crusts are very adherent; when they fall off or are displaced by emollient applications, round ulcerations are exposed, generally deep, with sharp and perpendicular borders, which are formed by a hard violet-coloured tissue; the bottom of the ulcers is greyish, dirty, and unhealthy; they have no tendency to increase in size; gradually the crusts again form, and fall off, until at last, by appropriate treatment, they become thinner, the ulcerations heal, and are replaced by sound and indelible cicatrices. This is the most common form of pustular syphilitic eruptions, and it is this which affects infants who are born with a syphilitic taint. In these cases the pustules are superficial, flattened, oval, and numerous; they are covered with blackish crusts, most frequently thin, and followed by small ulcerations; the physiognomy of such infants is very characteristic, their faces are marked with deep wrinkles, and they have the aspect of little old men; their skin is earthy, they are thin and blanched. Syphilitic pustules form in some cases around, in others beneath, the nails; they are followed by ulcers with a sanious discharge; the nails become detached, and are slowly re-produced, but they are thin, small, narrow, greyish, and friable.

4. *The Tubercular Syphilitic Eruption.* This is one of the most frequent forms of the venereal eruptions. The tubercles are of various sizes, of a red or copper colour, oblong, flattened, or conical, generally in groups, often forming circles: sometimes they remain for an indefinite time indolent, smooth, and polished; at other times they become covered with a slight desquamation, or they ulcerate. They most frequently form in the nose or in the commissures of the lips, so that a tubercle in one of these situations is almost a pathognomonic sign of a venereal affection. They may affect any parts of the body. Syphilitic tubercles present various appearances; the principal varieties are the following. Sometimes they are small, varying in size from the head of a pin to a pea, regularly arranged in small circles, the middle of which is entirely healthy; each tubercle is partially covered by a hard grey scale; they are of a coppery colour, rarely followed by ulceration. They leave a red livid stain, which eventually disappears. This variety is chiefly seen in the forehead and neck. The tubercles may be larger, oval, or pyriform, and very prominent, in irregular groups; sometimes they are as large as an olive, smooth and shining, never or very rarely terminating by ulceration or covered by exfoliations, or painful; they may remain stationary for years. It attacks the face, and chiefly the cheeks and extremity of the nose. In many cases the tubercles are large, isolated, rounded, few in number, of a violet red colour, surrounded by a coppery areola; they are most frequently developed in the face—on the upper lip and nose. Sooner or later they become painful and inflamed, and ulcerate; new tubercles are formed around the old ones, which go through the same course; the ulcers unite, and are covered by a hard, blackish, adhering incrustation; this when removed leaves an irregular ulceration, the borders of which are sharp and perpendicular, formed by a hard violet tissue; the crusts are renewed and the ulceration extends, sometimes destroying one ala of the nose, or eating away part of the lip. In the majority of those cases where the destruction of the bones, cartilages, and integuments of the nose has been complete, the disease has commenced in the bones and internal tissues, and has proceeded to the surface. In other cases the tubercles are large, red, hard, and rounded, dispersed over different parts of the body, and chiefly on the back; they are sometimes the size of a small nut, and they finally ulcerate. The ulcerations which are superficial and not many lines in breadth, spread in various directions, assuming sometimes a circular, at other times a spiral or a zig-zag form; healing at one extremity, and extending at the other;—they are covered with thick, hard, black, and very adherent crusts, and they leave indelible scars. Most frequently fresh crops of tubercles are constantly formed ; and as all do not ulcerate at the same time, they are seen in all their different stages on the same individual. Alibert described this as "syphilide pustuleuse serpigineuse." Tubercles assume occasionally another form; they are round, thick, and flat, and their surface becomes the seat of linear ulcerations. Sometimes these are not larger than a lentil, and form at the junction of the alæ nasi with the cheek, or at the commissure of the lips; at other times they are nearly the size of a shilling and some lines in thickness, of a deep livid red colour, occupying the scrotum, verge of the anus, or pubes; their surface becomes cracked, as it were, by linear ulcerations, which discharge a sanious and nauseous fluid.

5. *The Papular Syphilitic Eruption.—(Syphilitic Lichen.)* This is an eruption of small, hard, solid elevations, slightly raised above the level of the skin, containing no liquid, terminating either by resolution or desquamation, and never followed by ulcers or cicatrices. It may be acute or chronic. (1.) In the acute form the papulæ are very small and very numerous, slightly conical and of a coppery tint; in some parts the areolæ which surround the pimples, mixing together, give the skin the appearance of a large copper-coloured surface, studded with innumerable elevated points of a lighter colour. These papulæ may be found on any part of the body, but more particularly on the face; they are all found at the same time, and within 24 or 48 hours. Generally they are not accompanied by any feverish symptoms; they often accompany or immediately follow gonorrhœa, as Mr. Carmichael has observed. In the majority of cases their duration is short, and they terminate by resolution in a few days. It is possible that, as in lichen agrius, their summits may ulcerate; when this is the case, the crusts which form from the concretion of the discharge are small and thin, and are never followed by scars. (2.) When chronic, the papulæ are larger, flattened, the size of small lentils, and of a copper colour; their development is slow and successive ; they are very numerous and in groups, producing no itching. They occupy the limbs, especially in the direction of extension; also the forehead and the hairy scalp. They are frequently accompanied by other syphilitic eruptions, especially the pustular. Generally their duration is tedious; the summit of each papula becomes covered with a dry, greyish pellicle; this falls, and is as often renewed, until the elevations are reduced to the level of the skin, which for some time is marked by greyish white spots.

6. *The Scaly Syphilitic Eruption.* This is distinguished by dry greyish scales covering small copper-coloured elevations, terminating by resolution or desquamation, never by ulceration. In the greater number of cases it assumes the form of psoriasis, and particularly of psoriasis guttata. The eruption may be confined to one part of the body, but usually it is seen at the same time on the neck, back, breast, anterior surface of the abdomen, face, limbs, and sometimes on the hairy scalp; the size of the patches varies from that of a "centime" to a shilling; they are generally isolated, irregularly rounded, slightly elevated, covered by thin, hard, whitish, slightly adherent scales, which on falling expose smooth, shining, copper-coloured elevations, not red and cracked as those of psoriasis. When they resemble the patches of psoriasis guttata, each is surrounded by a small white border, exactly similar to that which indicates the remains of a vesicle: M. Biett regards this as a pathognomonic characteristic. In some rare cases the patches unite. That variety which Willan has described as lepra nigricans is considered by M. Biett to be a syphilitic form of lepra. The palms of the hands and the soles of the feet are sometimes attached by a very peculiar form of the scaly syphilitic eruption. The mass of scales is dry and horny, so that it can be completely detached by merely inserting the nail beneath one point of its circumference, and raising it a little; the surface beneath is not elevated, but is of a violet colour and indurated. This generally accompanies other syphilitic eruptions, especially the scaly.

The various secondary symptoms which occasionally accompany syphilitic eruptions are detailed; but as they are too well known, and are more fully examined in all works on the venereal disease, we shall not enter at all on the subject here. Syphilitic eruptions may be complicated with various cutaneous diseases, not depending on the same cause, as eczema, herpes, scabies, &c. Several of these different forms of syphilitic eruptions may exist at the same time on the same patient; the scaly variety is most frequently uncomplicated.

*Diagnosis.* 1. The exanthematous syphilitic eruption, when acute, may be confounded with roseola and urticaria—when chronic, with ephelis. The rosy colour, and the general symptoms which accompany roseola, distinguish it from the greyish patches of this syphilitic eruption. The colour of urticaria is either redder or whiter than the rest of the skin, never greyish; the patches of urticaria are more elevated, produce more intense itching, and they disappear suddenly, and again are spontaneously re-produced. When acute, this syphilitic eruption almost always accompanies the primary symptoms, and particularly gonorrhœa, or at least almost immediately follows them. The ephelides are larger than the chronic exanthematous syphilitic eruption; they are irregular and cover a larger surface of the body; they occupy especially the belly, and the anterior surface of the chest; the venereal patches are usually circular, rarely exceeding in size half-a-crown, few in number, principally situated in the forehead and eyebrows. The ephelides are yellow, attended with itching, and constantly covered with a furfuraceous exfoliation. The venereal patches are copper-coloured, sometimes blackish; they occasion no pruritus, and very rarely are covered by a slight desquamation; they never unite to form large irregular patches, as is the case with ephelides; they are almost always accompanied by some symptom of general venereal infection, often by iritis.

2. The principal diagnostic marks of the vesicular syphilitic eruption are the coppery areola, the slow progress of the inflammation, the preceding and especially the concomitant symptoms.

3. Pustular syphilitic eruption may be confounded with acne and ecthyma.

The pustules of acne differ from the psydracious pustules of syphilis, in being more prominent, red, and sometimes surrounded by a well-marked erythematous areola (A. rosacea); in the syphilitic eruption the pustules are more violet, and surrounded by a copper-coloured base. The skin which separates the pustules of acne is red, shining, oily, and interspersed with black points; it is earthy and shrivelled in the venereal eruption. The pustules of this latter affection are followed by scars; this is rarely the case except in acne indurata, which differs in many points from the other varieties. The phlyzacious pustules of the syphilitic eruption are, with some difficulty, distinguished from those of ecthyma; but, in ecthyma, the pustules are surrounded by a purple red areola; in the syphilitic eruption, it is invariably copper-coloured. The crusts of the latter are thicker and more adherent—sometimes almost black, having circular furrows, the ulcers beneath them are round, deep, with sharp and perpendicular edges, followed by a depressed and indelible cicatrix.

4. The tubercular syphilitic eruption may be confounded with lepra, some varieties of psoriasis, acne indurata, and lupus. In some cases previously described, syphilitic tubercles are arranged in circles, but these circles are not continuous as in lepra; the tubercles are smooth, small, copper or violet-coloured, covered with thin and hard lamellæ, always smaller than the induration to which they are fixed, whilst the scales of lepra are larger, extending beyond the elevated borders, so as to cover them, and sometimes the centre of the patch itself. The same characters will distinguish partially-cured syphilitic tubercles from psoriasis gyrata, for which they are often taken. The tubercles of acne indurata, which are constantly found on the back, mixed with numerous cicatrices, are often mistaken for syphilitic tubercles; but these latter, when situated on the back, are usually hard, rounded, and of a copper colour, often equal in size to a small nut; they usually ulcerate, and form the variously-convoluted creeping ulcers, very different, in the scars which they leave, to the small rounded cicatrix of acne. The early tubercles of lupus are sometimes with difficulty distinguished from those of syphilis; but in lupus they are reddish, soft, slightly developed, their summit is shrivelled and cracked, the skin surrounding them is swollen and œdematous; whilst syphilitic tubercles are more prominent, hard, smooth, shining, and of a copper colour. Lupus usually commences on the cheeks, tubercular syphilitic eruptions on the forehead and alæ nasi. The former attacks particularly the scrofulous, the lymphatic, and the young; the latter, persons of a more advanced age, and is almost always accompanied by other syphilitic eruptions, and by various secondary symptoms.

5. The papular syphilitic eruption may be confounded with scabies and lichen. The very small, slightly conical, syphilitic papulæ have been mistaken for those of itch; but the eruption of scabies is constantly vesicular—that of the other papular. The papulæ of lichen simplex are usually confined to one region of the body, and especially to the limbs; syphilitic papulæ cover the whole body, particularly the face; the former are not so deeply coloured, nor have they the violet areolæ.

6. The scaly syphilitic eruption may be confounded with lepra or psoriasis. The black colour of the syphilitic lepra (L. nigricans) sufficiently distinguishes it from the common lepra. The scaly syphilitic eruption may be mistaken, in some cases, for psoriasis guttata, the form of which it occasionally assumes; but the copper-coloured patches of the former, covered with thin, small, greyish scales, and surrounded by a narrow, white circular border, differ very considerably from the thicker scales of psoriasis guttata, which expose, in falling, a red and cracked surface. Independently of these more minute characteristic marks, the concomitant symptoms of secondary syphilis, as pains in the bones, nodes, ulcers in the throat, iritis, &c. most materially assist the practitioner in forming a diagnosis.

The incrustations which succeed pustules, or more frequently tubercles, may be mistaken for the scabs of impetigo; but in the latter the crusts are yellow, easily separated, merely, as it were, placed on the surface of the skin; in the former, they are greenish and almost black, sometimes marked with circular depressions, hard, adherent, and penetrating, more or less, the thickness of the skin. Syphilitic ulcerations may be confounded with those of lupus, but the former are deep and excavated; their borders are hard, callous, sharp and perpendicular, surrounded by a copper-coloured areola; the latter are superficial, or even raised, their borders are soft and of a violet colour, and the skin surrounding them is swollen, as if œdematous.

*Prognosis.* Venereal eruptions in themselves are not dangerous. The state of the patient warrants an alarming prognosis, only when they are accompanied by other severe secondary symptoms. The tubercular eruption is the most serious, but the scaly is generally very tedious.

*Causes.* In some cases, syphilitic eruptions are excited by errors in diet, severe exercise, mental agitation, &c.; in other cases, no such occasional cause can be discovered, but in every case the proximate cause is the venereal virus. They have been attributed to mercury; but patients are often covered with these eruptions who have never taken a grain of that mineral. In certain states they are contagious; they may be hereditary; thus infants at birth have been covered with syphilitic pustules, or the eruption has formed some little time after birth. In other cases, the infection is conveyed to the child by means of the milk of its nurse; and frequently the infant infects the nurse. M. Biett considers that these eruptions may follow gonorrhœa, as well as primary venereal sores, buboes, &c.

*Treatment.* Mercurial preparations are the most useful remedies that can be employed in the cure of syphilitic eruptions. Their failure is generally owing to their injudicious administration; they should never be given if the symptoms are acute, and the dose should be regulated by the strength of the patient and the extent of the disease. In general, the liquor of Van Swieten (Oxymur. hydrar. gr. xvj. Alcohol. ℥ss. Distilled water, ℔j.—Dose, half an ounce night and morning in milk, solution of gum, or water), or the following pills may be employed.

℞. Hydrargyri oxymuriatis. . . . gr. xij.
Extr. aquos. opii . . . . . . . . . ℈j. Misce et divide in pilulas xxxvj. quorum æger sumat j. omni die.

If the patient is irritable and weak, so that there is a probability of the mercury's producing irritation of the gastro-intestinal mucous membrane, a pill, containing a grain of the soluble mercury of Hahnemann (black oxide of mercury), may be given daily. Mercurial preparations, judiciously administered and in small doses, are rarely injurious; if symptoms of abdominal irritation should come on, their use should be interrupted for the time.

To guard against relapse, the medicine should be continued for a month or more after the eruption has disappeared. Sudorifics, particularly when combined with other remedies, are often very useful, as the decoctions of guaiacum or sarsaparilla. The ptisan of Feltz has sometimes been very beneficial, especially when mercury has failed; the following is the formula.

℞. Radicis sarsaparillæ concisæ. . ℥iij.

Icthyocollæ . . . . . . . . . . . . . . . ʒss.

Pulv. antimonii crudi . . . . . . ℥iv.

Aquæ fontanæ . . . . . . . . . . . . . ℔vj.

The antimony to be inclosed in a bag of muslin, and the whole boiled gently until the water is reduced to one-half. A pint and a half is the proper daily dose. M. Biett, in some cases where mercury has failed, has succeeded in curing the eruption by full doses of subcarbonate of ammonia; he commences with the daily dose of a drachm of the salt in some mucilaginous draught, and he gradually increases it to two or three drachms. He has found it advisable to hasten the resolution of the tubercles, by gently rubbing in with the end of the finger an ointment, composed either of a scruple to half a drachm of the proto-nitrate of mercury, or one scruple of the proto-ioduret, or 12 grains of the deuto-ioduret of mercury to an ounce of lard. For the same pupose, but with still more beneficial effects, M. Biett has prescribed the ioduret of sulphur, in doses of from 20 to 30 grains to the ounce of lard.

It is sometimes necessary to endeavour to produce a new action in syphilitic ulcers, or to stop their destructive progress; for this purpose, stimulating ointments, containing the deutoxyde, the deuto-ioduret, or the cyanuret of mercury, may be applied; or more energetic caustic applications, as the acid nitrate of mercury, applied by means of the feather of a quill to the diseased surface. M. Biett has found that the severe pain of these ulcers has been soothed by the application of an ointment, made by mixing 20 drops of medicinal hydrocyanic acid with two ounces of cerate. The action of these various remedies may be assisted by baths; vapour douches, directed on the tubercular eruption for 12 or 15 minutes daily, hasten its resolution: alkaline baths are to be employed in the pustular eruption, and vapour baths in the scaly. Those large flat tubercles, which have been described as forming on the scrotum and verge of the anus, are improved and cured by cinnabar fumigations. Occasionally syphilitic eruptions, particularly when complicated with other alarming secondary symptoms, resist every treatment which has been recommended, but give way to opium, in large doses. M. Biett commences with half a grain of the watery extract of opium, and gradually increases it, if necessary, to four grains, or even more.

If an infant, before it is weaned, is attacked by syphilitic eruptions, the mother or nurse who suckles it should take the liquor of Van Swieten, or what is better, should rub into her legs and thighs camphorated mercurial ointment. If the person who suckles the child is too weak to adopt this treatment, the infant should take the milk of a goat which had been similarly rubbed with mercurial ointment.

We have thus compressed into a few pages the pith of an article extending to nearly sixty, and laid before our readers a comprehensive view of a class of eruptions second in importance to none, although usually discussed by surgical writers, under whose care they fall, in an unsatisfactory and superficial manner. On turning to the various surgical lectures, published by the weekly medical journals, and professing to be verbatim reports of these productions of the most celebrated lecturers of the day, it will be found that the descriptions and treatment of venereal eruptions occupy but two or three pages, and that the only diagnostic marks given are the copper colour, the form of the ulcers, and the concomitant symptoms: it cannot be that the teachers are not fully aware of the importance of the diagnosis of these eruptions ; but from having been in the daily habit, in hospital practice, of seeing large numbers of cases themselves, they are able, at the first glance, to decide on the nature of the disease ; and forgetting that this " visus eruditus" was not intuitive, they consider that what is so evident to themselves must be as clear to others, who have not had the same means of obtaining information. The French, in general, run into the opposite extreme; and, either from giving their pupils no credit for observation or judgment, or from their finding it impossible to keep to themselves any information they may possess, their clinical lectures and books too often abound in elaborate descriptions of the most trivial matters. Roux was struck with the difference, which every one must have observed, in the modes of communicating instruction in the two countries; and he has dexterously excused the national vanity of his own nation by quoting the declaration of Seneca, that he would consent to know nothing if his knowledge was to be for himself alone, if he were forbidden to communicate it to others, to prove that the failing is at least *classical.* We are glad to find that the articles on cutaneous diseases in the Cyclopædia of Medicine, which have been confided to the able hands of Dr. Todd, are likely to supply, in some measure, the deficiencies of the works of Bateman and Plumbe in this class of diseases. He has judiciously determined to follow up the arrangement which both Willan and Bateman evidently intended to have pursued, in classing the various syphilitic eruptions according to their external characters, and not confounding them together in one group. Dr. Todd, in the article " Acne," gives as a strong reason for this proceeding, that although certain forms of cutaneous disease appear essential to syphilis, yet the greater number of those described as syphilitic depend on the constitutional predisposition of the individual, modified by the syphilitic virus ; and that " if lichen, lepra, or any other cutaneous disease, makes its appearance in persons tainted with the venereal poison, these diseases, though originating in ordinary causes, will suffer a relative modification." We suspect that this is rather a pathological speculation than the result of actual experience. How frequently is itch complicated with chancres, buboes and venereal eruptions, and yet it suffers no modifications, but yields as readily to sulphur as the most simple case. We recollect to have seen, in the Hôpital des Venériens, a young man who was affected with both psoriasis guttata and the scaly syphilitic eruption; the former most abundant on the legs, the latter on the trunk, the smooth, polished, coppery, syphilitic eruptions having on their surfaces small, white exfoliations, contrasted in the most marked manner with the redder, rougher, and cracked surface of the psoriasis, with its thicker scales. The psoriasis g. was of some years' standing, but its appearance was not in the least modified by the syphilitic taint. Our authors mention that they have seen many times both herpes and eczema complicated with venereal eruptions. Since the opinion of Hunter and Abernethy, that true syphilis cannot be cured except by mercury, has been completely disproved by the extensive experiments of Mr. Rose, we know of no test by which Dr. Todd can prove the existence of "certain forms of cutaneous disease essential and peculiar to syphilis," distinguishing them from those generally described as syphilitic.

### Purpura.

M. Biett has judiciously, in this instance, departed from the classification of Willan, who placed this eruption under the order "Exanthematæ;" for the exanthematous eruptions are produced by inflammation and injection of the cutaneous vessels; whereas, in purpura, there is neither inflammation nor injection of these vessels, but an effusion of blood in the superficial layers of the dermis.

*Purpura Simplex.* M. Biett has found that in those cases in which, from the constitutional debility of the patient, tonics are indicated, benefit has been derived from hot alcoholic fumigations to the part; their temperature should not be elevated beyond 104° to 110° Fahrnheit.

*P. Hæmorrhagica.* Nothing is added here to the excellent description which Bateman has given of this species, nor have our authors at all cleared the obscurity of its etiology. In the examinations of the bodies of those who have died of this disease, the purple spots and the ecchymoses are found to be formed by effusions of blood in the cutaneous and subcutaneous tissues; these are easily washed off, and no increased vascularity at these parts can be detected. Similar effusions are frequent beneath the mucous membrane of the mouth, pharynx, stomach and intestines—more rarely beneath the peritoneum and pleura; they have been found beneath the pericardium, on the surface of the heart. Generally there are effusions of blood into the tissue of the lungs, constituting pulmonary apoplexy. In some cases, there are partial effusions in the muscles and in the viscera. In a fatal case which occurred in the Hôpital St. Louis, there were effusions of blood in the brain, lungs, liver, spleen, kidneys, in fact in almost all the organs.

Although the general debility which accompanies this eruption would appear to require tonics, wine, full diet, &c. yet this treatment has not been found to succeed, unless in the aged or with children, or with those who have been debilitated by bad nourishment or excessive fatigue. M. Biett has found that the most successful treatment, even in the severe cases, consists in giving the patients acidulated and laxative drinks; with some, he has employed with advantage the extract of rhatany, with ice. The patient should breathe a pure air, and live in a dry and open situation; his diet should be mild, composed of animal jellies, and a small quantity of roasted white meats, with good light wine, diluted with water and iced.

### Elephantiasis of the Arabs.

This, which has been confounded with elephantiasis of the Greeks, a tubercular disease which we have before mentioned, is characterized by a tumefaction of the skin, and of the subjacent adipose and cellular tissue, hard and permanent, causing frequently a deformity fully justifying the name which is applied to it. From its being endemic in Barbadoes, it has also been called "the Barbadoes leg." It most frequently occupies one of the lower limbs, but it may attack any part of the body. M. Alard, in 1806, published an excellent monograph on this disease, which he attributed to a frequently-recurring erysipelatous inflammation. Generally, it comes on with pain and swelling in the tract of the lymphatic vessels and glands, the skin is attacked with erysipelatous inflammation, the cellular tissue becomes inflamed and tumefied; the constitution often sympathizes; there is fever, thirst, nausea, vomiting, sometimes delirium; these symptoms, with the exception of the slight tumefaction, subside, but frequently recur, leaving after each attack an increase in the enlargement and in the hardness of the part. Thus the disease proceeds until it has produced considerable deformity, and it then may remain stationary for many years. The tumefaction of the limb may be uniform, entirely disguising the natural shape of the part; or it may be unequally enlarged, the different swellings being separated by deep fissures. The tumefaction may gradually extend from the leg to the thigh, and from the forearm to the arm, or it may be confined to the back of the hand or foot; the palms and soles never participate in the tumefaction. The skin sometimes is merely whiter and more resisting; at other times it appears violet, from the enlargement of the subcutaneous veins: but it may be attacked with vesicular inflammation, and become covered by thin, soft, yellowish laminæ, or even harder scales, analogous to those of icthyosis, or by small fungous granulations; finally, there may be fissures or ulcerations, covered by thick yellow crusts. M. Alard has described analogous tumefactions of the skin and cellular tissue covering the neck, chest, abdomen, &c. but these cases are very rare. The penis is more frequently enlarged by this disease; M. Biett has seen one case in which its volume was quadrupled. The teats, in some instances, are so increased in size, that it is necessary to suspend them with bandages passed behind the neck.

*Causes.* It is neither contagious nor hereditary. It attacks most frequently adults, but may be met with in infancy and old age. It is endemic in the Torrid Zone; it is rare in Europe. We believe that Dr. Graves, of Dublin, first attracted the attention of the profession to the frequency of this disease in Ireland: as physician to the Meath Hospital, he has had many opportunities of watching the progress of this form of elephantiasis, and the results of his observations he has published in the 4th Volume of the Dublin Hospital Reports; the history of the progress of the disease fully confirms the description which we have given; indeed, it is all but a literal translation of that published by Alard, in 1806; but as it is not marked as a quotation, we presume that Dr. Graves was unacquainted with the work. From inquiries which he caused to be made in the South of Ireland, he found that the disease was by no means rare there; that men, women, and children are all subject to its attacks, and that some have laboured under it from infancy to extreme old age; it is also comparatively very frequent in the Dublin Hospitals. Dr. Graves describes no other form than that attacking the upper and lower extremities. Considerable enlargement of the labia majora, from this affection, is not unfrequently seen affecting prostitutes; and we recollect a woman who went the tour of the London Hospitals, with enormous, hard tumefaction of the skin and cellular tissue of the perineum, verge of the anus and lower part of the nates, resembling in every particular this affection. Our authors allude to the prodigious enlargement of the teats from elephantiasis in some rare cases. If Juvenal, in the following lines, alludes to this affection, it must have been as common an endemic in a city in Ethiopia, as goitre in Switzerland,

> Quis tumidum guttur miratur in Alpibus ? Aut quis
> In Meroë crasso majorem infante mamillam ?—Satire 13th.

From the pathological researches of Chevalier, Andral, and Bouillaud, it appears that the epidermis is very thick and adherent; the rete mucosum distinct, and can be separated into several layers; the papillæ elongated and enlarged; the corion so much hypertrophied, that in some cases it is half an inch in thickness; the cellular tissue considerably developed, sometimes containing a gelatinous fluid, but more frequently of almost a scirrhous hardness, being more dense the nearer it is to the skin; the muscles pale, softened, and thin ; in some cases the veins are obliterated.

*Treatment.* During the inflammatory stage, the antiphlogistic treatment is to be pursued—either general bleeding, or leeches to the neighbourhood of the inflamed lymphatics. In the chronic stage, general and local bleeding, scarifications, blisters, the cautery, and mercurials, have been tried, but without success. The remedies which appear to have produced the greatest impression in this disease, in the Hôpital St. Louis, are compression, friction, and vapour douches. Gradual and equal compression of the whole limb should be made by a roller, three or four fingers' breadth in width ; this either rapidly diminishes the tumefaction, or facilitates the action of other remedies ; frictions should be made, with an ointment composed of half a drachm of hydriodate of potash and an ounce of lard ; this should be omitted the moment there is any appearance of inflammation. Vapour douches should be directed against the tumefied parts for a quarter of an hour daily. If the skin is inflamed, or covered with vesicles, emollient applications and warm baths must be substituted.

### Disease of the Sebaceous Follicles.

M. Biett has frequently observed, in his wards, an inflammatory state of the sebaceous follicles of the skin, sometimes attended with an increased exhalation of the unctuous fluid, and at other times by a marked alteration in its quality, as well as quantity. The follicles of the face are most frequently affected, but the whole system may be inflamed. At first there is merely increased secretion, giving the parts an oily appearance, without changing the colour of the skin ; this secretion augments in quantity and thickness, and eventually forms a scaly lamina, which is soft and easily removed ; this gradually becomes harder and more adherent, the skin beneath it is red, and, on examination with a lens, the openings of the sebaceous glands appear dilated, and sometimes obstructed by the hardened secretion. This layer may be detached by profuse perspiration, or it may remain for months, growing dry and black. This inflammation rarely extends to the other tissues of the skin ; in some rare cases, the secretion has the appearance of the sero-purulent fluid contained in the vesicles of eczema. This inflammation may remain some weeks, or even years.

*Causes.* It is confined to youth and adult age, and to those whose skin is naturally white, thin, and unctuous; it has never been observed in infancy or old age. Young and lymphatic women are sometims the subjects of this affection after parturition. Exposure to a cold wind has produced it.

*Diagnosis.* If the inflamed follicles are numerous, and spread over a considerable extent of surface, and the hardened secretion forms a thick, blackish layer, so divided as to appear like a mass of imbricated scales, it may be taken for icthyosis; but, in this latter affection, the scales are deeply implanted in the skin by one of their borders; they are dry, very adherent, and, in order to be detached, must be plucked out; this is never the case with the scales produced by the hardened secretion of the inflamed sebaceous follicles.

*Treatment.* When the inflammation is circumscribed, M. Biett has found that it would yield, in a few weeks, to the vapour douches, directed on the diseased surface for 15 or 20 minutes daily, and to the employment of narcotic lotions, succeeded by astringent washes, containing alum, or the vegetable acids. Mild purgatives may be employed when there are no symptoms of gastro-intestinal irritation, which is often the case when the inflammation of these follicles occupies a large surface of the skin; when this occurs, the layers are to be detached by mucilaginous or gelatinous baths and gentle friction—subsequently, alkaline baths, with slightly stimulating applications and vapour-baths. The diet should be mild.

### KELOIDE.

This was first described by Alibert under the name of cancroide. Bateman, who had never seen it, doubted its existence; but M. Biett has had several cases which have confirmed the accuracy of Alibert's description.

Kéloide commences by slight tumefactions of the skin; these increase in size, and form small flattened tumours, generally of an oval form, with a slight central depression; at other times they are elongated and angular; the epidermis covering them appears thinned and slightly wrinkled, like the cicatrix of a deep burn; the tumours are hard and resisting; their colour is sometimes a deep red, at others of a pale rose tint, changing according to the temperature, and in women at the period of menstruation; they project one or two lines above the level of the surrounding skin, their circumference being more elevated than their centre; they may increase to an inch and a half to two inches in diameter, or not exceed a few lines. M. Biett has seen a young woman who had eight of these tumours in her neck and anterior part of the chest. In some cases there is deep and lancinating pain, especially after eating, or during atmospheric changes, but this is not invariable; some are never attended with pain. The progress of kéloide is slow—it very rarely, if ever, terminates in ulceration; in some cases it becomes absorbed, and a white and firm cicatrix remains. It has been most frequently observed on the anterior part of the chest, sometimes on the neck and arms.

*Causes.* Owing to the few cases which have been observed its etiology is obscure. In some the tumours formed without either local or general derangement, in other instances they followed an injury. It has generally been observed in young persons, never in infants.

*Diagnosis.* Kéloide should be carefully distinguished from a cancerous affection of the skin; the latter is known by its prominent, round, and violet tubercles ulcerating at the summit, and surrounded by dilated veins; the neighbouring glands are tumefied, whilst the tumours of kéloide, which are generally situated on the anterior part of the chest, are prominent, flattened, with elevated borders, and generally covered with healthy skin.

*Prognosis.* It is never a serious disease; generally the subjects of it have been in excellent health.

*Treatment.* Extirpation and cauterization are not advisable. Sulphur douches sometimes have softened the tumours. Probably frictions with an ointment containing hydriodate of potash would be found advantageous.

We cannot close this article without recommending the work which we have analyzed, as a valuable companion to Bateman's Synopsis. M. Biett, whose clinical lectures form the ground-work of this compilation, adopted the classification of Willan, instead of the more superficial system of Alibert, from a conviction of its excellence; this conviction has increased with his experience, and although he has added facts, and has thrown considerable light on diagnosis, he has not found it necessary, except in the most trifling details, to alter the clear and accurate arrangement of our countryman. This testimony, from a foreigner, and particularly from a physician of M. Biett's talents, and ample experience in favour of the classification of Willan, is of the highest value. Unless a clear knowledge is obtained of the various forms which cutaneous diseases assume, often so similar in appearance, but so dissimilar in the remedies which they require for their cure, no definite rules for treatment can be applied. That the acquisition of this information is materially assisted by an accurate classification is evident from the imperfect acquaintance we possessed with these diseases prior to the time of Willan, and pathologists must award due praise to those who further this object. The English students who have attended M. Biett's practice and his clinical lectures, and have experienced the uniform kindness with which he receives the numerous foreigners, of all nations, who crowd his wards, will bear willing evidence, not only to his minute acquaintance with every form of cutaneous disease, but to his skill in applying remedies, and to the success which follows them.

